# Fermented mutton in the Faroe Islands: the survival of a local artisanship and food heritage

**DOI:** 10.1186/s42779-023-00182-7

**Published:** 2023-06-05

**Authors:** Ingvar Svanberg

**Affiliations:** Institute for Russian and Eurasian Studies, Uppsala University, Box 514, 751 20 Uppsala, Sweden

**Keywords:** Biodiversity, Gastronomic ethnobiology, Grassroot movements, Heritage food, Island foodscape, Local knowledge, Microbial communities, Meat preservation, Nutritional anthropology, Tourism

## Abstract

Dried and fermented mutton has been an essential storable protein source in an economy where weather conditions and seasonal fluctuations affect the availability of food. For generations, the Faroe islanders have prepared *ræstkjøt* (fermented and semidried mutton) and *skerpikjøt* (dried mutton) as an efficient and valuable cultural strategy for preserving meat. The data for this study have been collected through anthropological and ethnobiological fieldwork as an embedded participant observer, supplemented with studies from written sources. Data were selected and qualitatively analysed. Our findings show that this traditional cuisine, so far rarely noticed by researchers as a food heritage, requires that the islanders have access to sheep, master the technique of properly treating the slaughtered carcases, and that the necessary ecological conditions, in relation to wind and temperature, prevail for the meat to ferment and dry. They must also have access to the necessary equipment and skills, and be able to assess when the dried meat is cured. The relationship that exists between humans and the active microorganisms in this specific context is also discussed. Appreciating and consuming local fermented food is also an important way of expressing Faroese cultural identity. Once a staple for rural people, fermented mutton is nowadays a rather exclusive delicacy. The study provides insights into a complex activity that includes local artisanship and food heritage based on the triangle of human–sheep-microbiota.

## Introduction

Contemporary Faroese cuisine offers a wide range of interesting dishes based on locally available mammals (sheep, small cetaceans), as well as seafowl and fish. All of these easily qualify as heritage food that is still of great importance in the islands’ foodscape. Cooking methods have, however, in most cases changed over time by adapting to contemporary taste preferences, and the availability of new flavourings and technical equipment [1].

Faroese dishes can be consumed raw, dried, fermented, cooked, or fried [2].Old-fashioned conservation methods using natural factors, such as air temperature, humidity, and wind, are important, as is the diversity of microorganisms. Wind-dried mutton which has undergone various stages of fermentation, a process known as *ræst* (adj.), are highly prized foods. The most cherished and popular delicacy is *skerpikjøt*: well-aged, wind-dried mutton, which is quite chewy [3]. Earlier, salt was a precious commodity and therefore not used for the preservation of meat [2, 4].

**Fig. 1:**
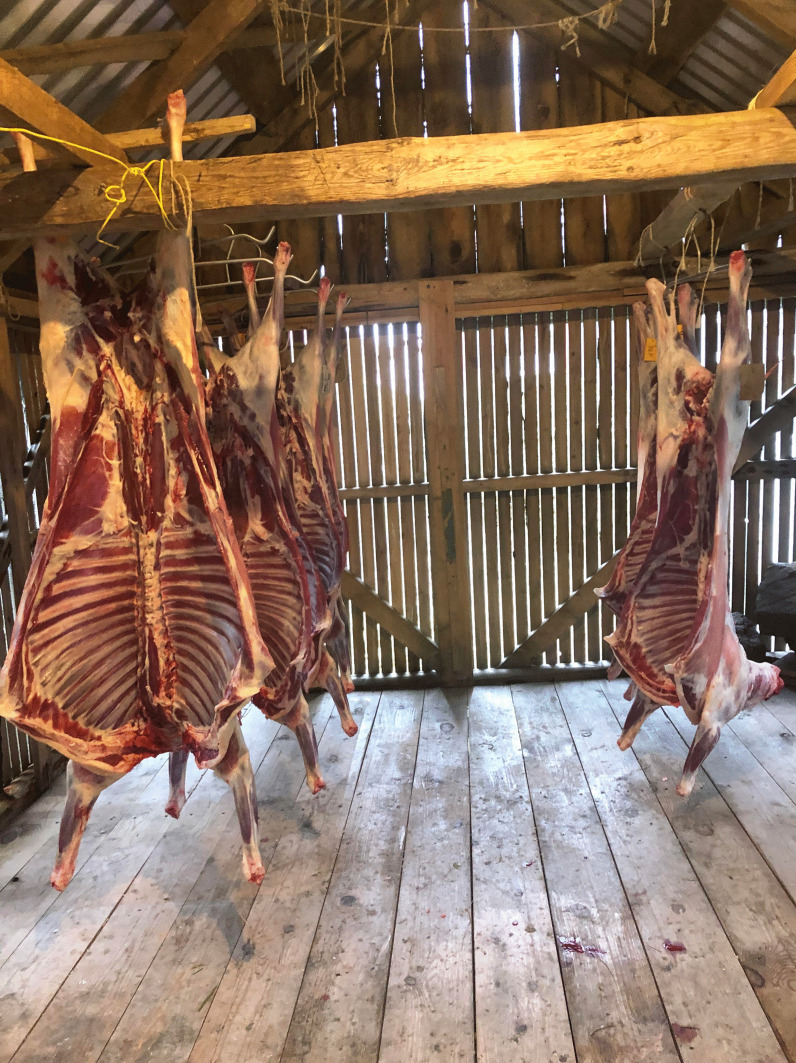
Newly slaughtered sheep drying in a *hjallur* in Gjógv, Eysturoy, 2020 (Photo Navarana Ingvarsdóttir Olsen, October, 2020)

This foodway requires access to sheep, and a mastery of the technique of properly treating the slaughtered sheep carcase. It also depends on the occurrence of the right conditions, like wind and temperature, for the meat to ferment (Fig. 1). The islanders’ knowledge of the resources is essential for making and maintaining these foods [5].

Local food is produced within a specific cultural context and based on interaction between local communities, the natural environment, and its biota. As ethnobiologists, we study how a specific group of people uses the biodiversity of the environment, where its transformation into food and energy is fundamental [6]. We can distinguish between *diet*, i.e. the food people consume; *subsistence*, i.e. the way in which the provisions are acquired; and *foodways*, i.e. the entire set of activities, symbolism, and beliefs surrounding the acquisition, preparation, and serving of food [7]. The availability of the local diet depends on ecological conditions, which vary over time. Social-ecological systems are complex when it comes to subsistence and involve many factors [8]. Some contemporary diets, especially traditional meat dishes, have high cultural, emotional, and nutritional value [9].

During the past few years, several ethnobiologists have discovered fermented food as an interesting topic [10–12]. The systematic study of local food production and consumption is central to many ethnobiological research projects, since it provides us with good insights into the complex activity context between animal and human culture, and the bio-cultural domains that emerged from these relations [9].

In the circumpolar area, fermentation has been a common way of preserving meat [13, 14]. However, in the Nordic countries this way of preparing meat has increasingly disappeared from the cuisine. In the Faroe Islands, it still exists as an important part of foodways [2, 5]. Fish—mainly cod, *Gadus morhua* L.—are fermented and eaten as everyday food, known as *ræstur fiskur* [2, 5]. Also, meat of the pilot whale, *Globicephala melas* (Traill), is eaten dried and fermented [15].

However, the most prestigious dishes in the Faroese diet are made from air-dried and fermented mutton. This is not only a result of an activity context between humans and sheep, but also about the relationship that exists between humans and the active microorganisms in this specific context.

## Environmental, ethnographic and social framework

**Fig. 2:**
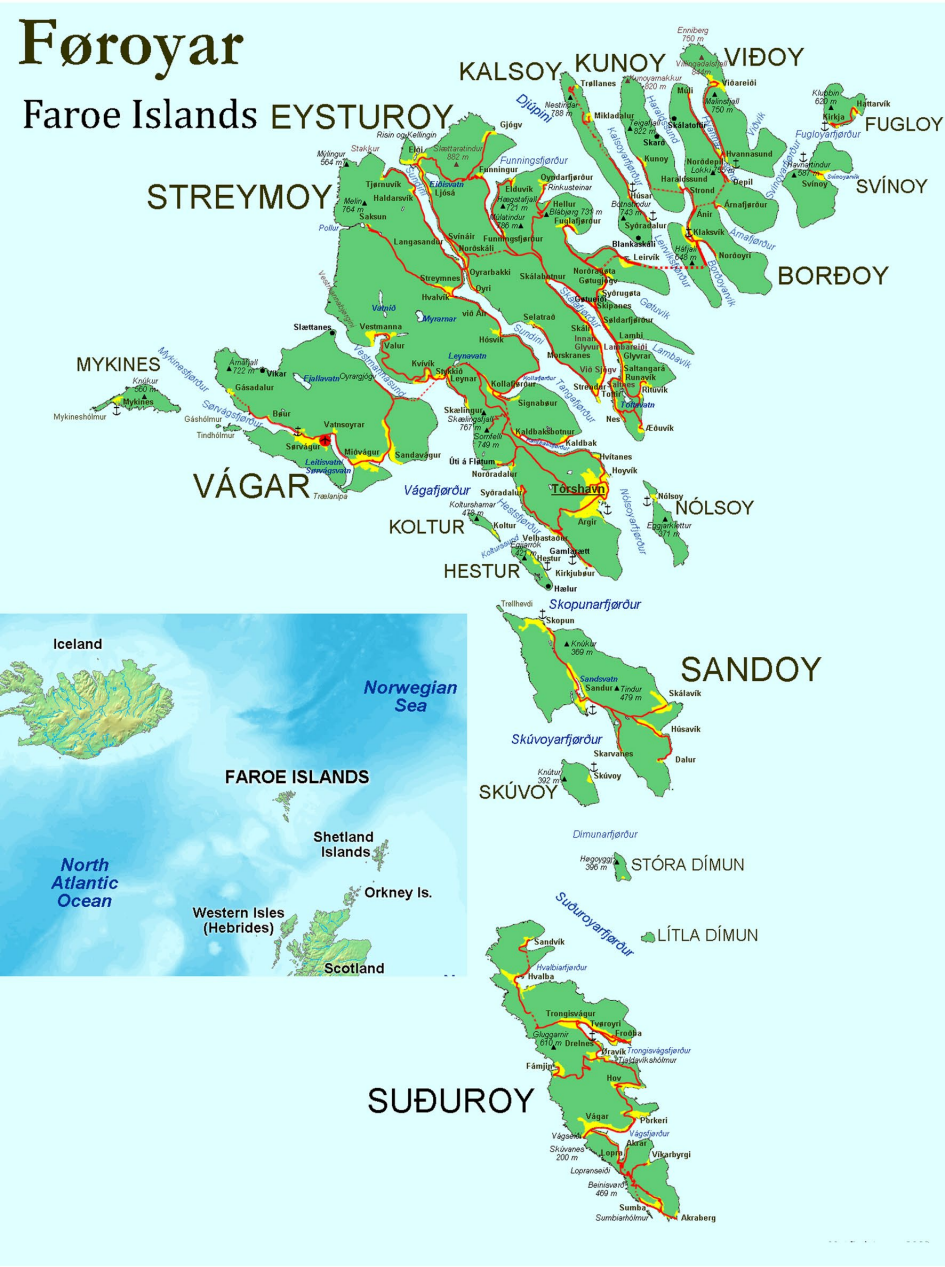
Map over the Faroe Islands (Source: Wikipedia CC BY-SA 3.0)

The Faroe Islands consist of 17 populated (out of 18) islands, with dramatic nature, and unpredictable weather. They are located in the North Atlantic Ocean, at 62° N 7° W, and lie almost midway between Norway, Iceland, and Scotland. The area constitutes around 1400 square kilometres. The climate is subpolar oceanic, influenced by the North Atlantic Current. Winters are mild and summers cold. It is windy and rainy all the year around [16] (Fig. 2).

There are traces of early colonizers from the sixth century. Fragments of sheep DNA and chemical residues of sheep faeces indicate that these first settlers brought domestic animals to the islands [17]. In 825, the Irish monk Dicuil described the islands as ‘full of countless sheep’ [18]. However, archaeological evidence indicates the contemporary Faroe islanders are descendants of Norse Vikings that settled in the islands later, in the ninth century [18].

The current population is 53,630 inhabitants (2023). About 40 per cent live in the capital, Tórshavn [19]. The majority of the population speaks the Faroese language, a Nordic language originating from Old Norse. Most people are bi-lingual, speaking and understanding Danish, which is taught in schools as a compulsory second language. Culturally, the contemporary Faroe Islands comprise a small and relatively homogenous society. Socio-economically, the Faroe Islands are today a modern welfare society with a high standard of living, on a par with other Scandinavian countries, and with almost full employment (the unemployment rate is insignificant), and high wages [16].

**Fig. 3:**
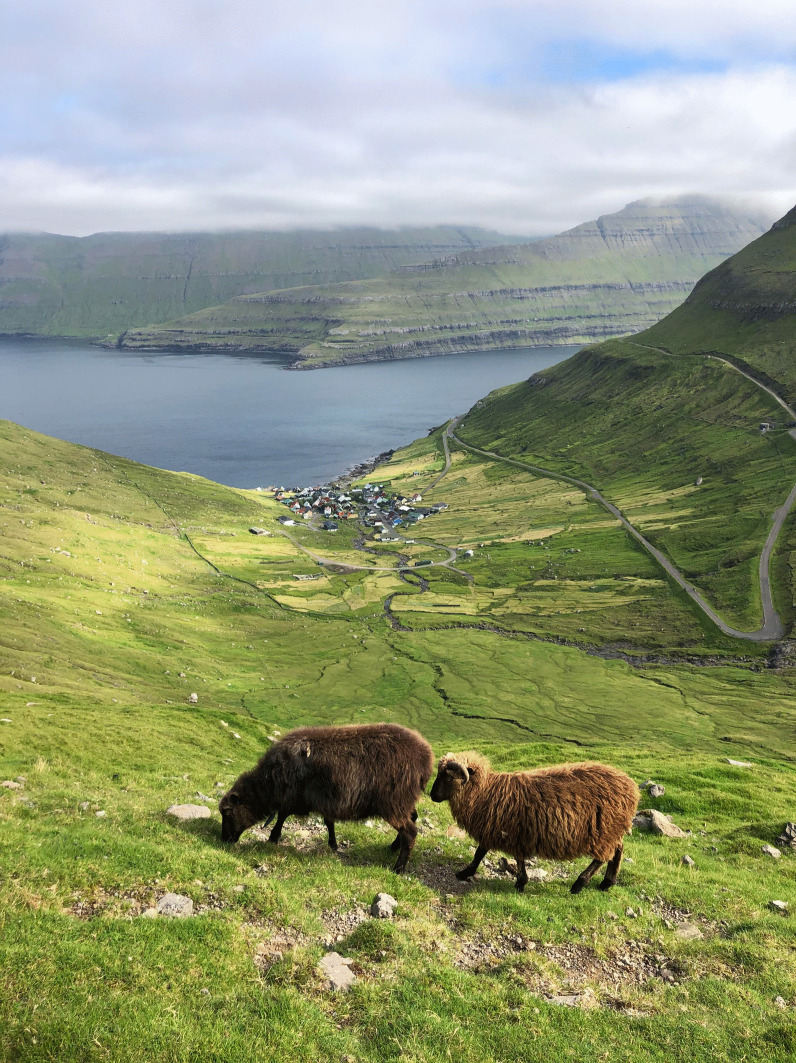
Pasturage for sheep outside Funningur, Eysturoy, August 2020 (Photo Navarana Ingvarsdóttir Olsen)

Most Faroese villages are at the foot of steep, grass-covered slopes. The actual village area is called *almenningur* (‘common land’). Around the houses are the cultivated infields, *bøur*, mainly used for hay production. Further off are the outfields (*hagi*) where the sheep graze [20] (Fig. 3) Sheep husbandry, seabird fowling, small-scale fishing, whale hunting, and some barley cultivations were the main means of economy since the settlement and for many centuries [21–23]. Production of knitted stockings and other woollen goods were important trade products from the seventeenth to nineteenth centuries. Knitting retained its importance in supplying fishermen with clothing into the twentieth century [24, 25].

Denmark maintained a monopoly over trade with the Faroe Islands and forbade the inhabitants from trading with others. When the Danish monopoly was abolished in 1856, an export-oriented commercial fishing industry rapidly developed in the Faroe Islands, kick-starting the development of a modern market economy and population growth. This led to the modernization of the society and greater integration into the world economy, resulting in many non-local food items becoming available in the stores [26, 27].

Over the generations, the ecological conditions and the relative isolation of the Faroe Islands have reinforced a strong cultural identity and a reliance on local foods. The economy depends almost entirely on exports of fish and fish products, which in 2020 accounted for 90 per cent of exports by value and comprised 20 per cent of the GDP [16].

Nowadays, most foodstuffs (with the exceptions of fish, seabirds, whale meat, some mutton, and potatoes) are imported to the islands. Some horticulture occurs, with small-scale production of rhubarb (*Rheum rhabarbarum*), blackcurrants (*Ribes nigrum*), as well as some gooseberries (*Ribes* *uva-crispa*) [28]. Potato cultivation was introduced in the mid-nineteenth century, and potatoes are still grown for household consumption in the villages [29].

The majority of the cultivated land on the Faroes has always been worked for the production of hay, which has been fed to sheep during the winter season. Hay is the only crop that remains important today [30].

Agriculture, once the dominant occupation, now contributes less than one per cent of the national production [19]. Although there are 70,000 sheep on the islands, they provide only 40 per cent of the total mutton consumption. The remainder is imported from the Shetlands, New Zealand, and Iceland. The Faroese sheep farmers believe that the special fermented mutton can only be made from the local breed of sheep, which have grazed on the hillsides and stiff cliffs eating the native grass. Imported mutton is never fermented [1].

## Objectives, sources, and method

In this article, we will discuss the importance of fermented mutton in Faroese cuisine, its history, its cultural impact, and its symbolic value in the modern context. Data for this study were gathered through yearly fieldwork in the Faroe Islands since the summer of 1994 until 2022.

Since the mid-1990s, I have on an annual basis collected field data through the anthropological and ethnobiological method of fully embedded participant observations. The interviews with informants have been unstructured and conducted according to the generative method, which is based on the respondents’ own point of view. By using these methods, my objective has been to record local knowledge about Faroese diet, subsistence, and foodways [1, 31]. The interviews and participant observation took place over an extended period of almost 30 years especially in Vestmanna, Gjógv, and Tórshavn (the capital of the islands), although I have visited many villages since 1994. Through the observation method, I learned about the activities connected with food as well as local knowledge about the environment of the people in their natural setting. In addition, as an observer, I was introduced to various activities within the human food chain, from slaughtering to cooking and consuming. I have taken part in the making most of the dishes mentioned here [1, 5]. My hosts also taught me about a landscape, which, in their eyes, was still able to support them with many necessities [32]. This was true, although they hardly used the resources anymore, with the exception of their dependency on fish (and occasional hunt for pilot whales) from the marine biota, harvest of seabirds, and hay production and sheep grazing on the terrestrial biota, which included not only local ecological knowledge but also rules for mutual help, sharing, and social networking.

The research has centred on ethnozoological issues [1, 5, 33]; plant knowledge has also been included in the data collecting [28, 34]. The ISE Code of Ethic Principles was followed in the fieldwork [35].

Supplemental data about historical culture and foodways were gathered from ethnographic reports, historical accounts, public health studies, and travelogues [36]. Data were selected from these written sources and analysed qualitatively. The historical review is important for our understanding of the past and present use of fermented meat in Faroese foodways. Unfortunately, very few statistical data on food consumption are available in the Faroe Islands, both at national and household level. This applies in particular to household-produced food such as dishes made from mutton, fish, seabirds and whale meat [19].

## Local knowledge and shepherding

The name Føroyar is believed to derive from Old Norse *Færeyjar*, which literally means Sheep Islands [37]. Until the late nineteenth century, the islands can be described as having a peasant society highly dependent on sheep husbandry [36]. The cattle population on the islands has not had the same cultural significance as the sheep, although they were kept for milk production [1, 36]. In Viking times, the sheep were kept on summer pastures in the mountains, but the shielings were phased out during the eleventh and twelfth centuries, when sheep grazing in the outfields developed [38]. Fish, seabirds, and marine mammals were important elements of the local diet [38]. While pig breeding ceased by the thirteenth century, keeping sheep has continued to be of importance for the islanders to the present day [39].

**Fig. 4:**
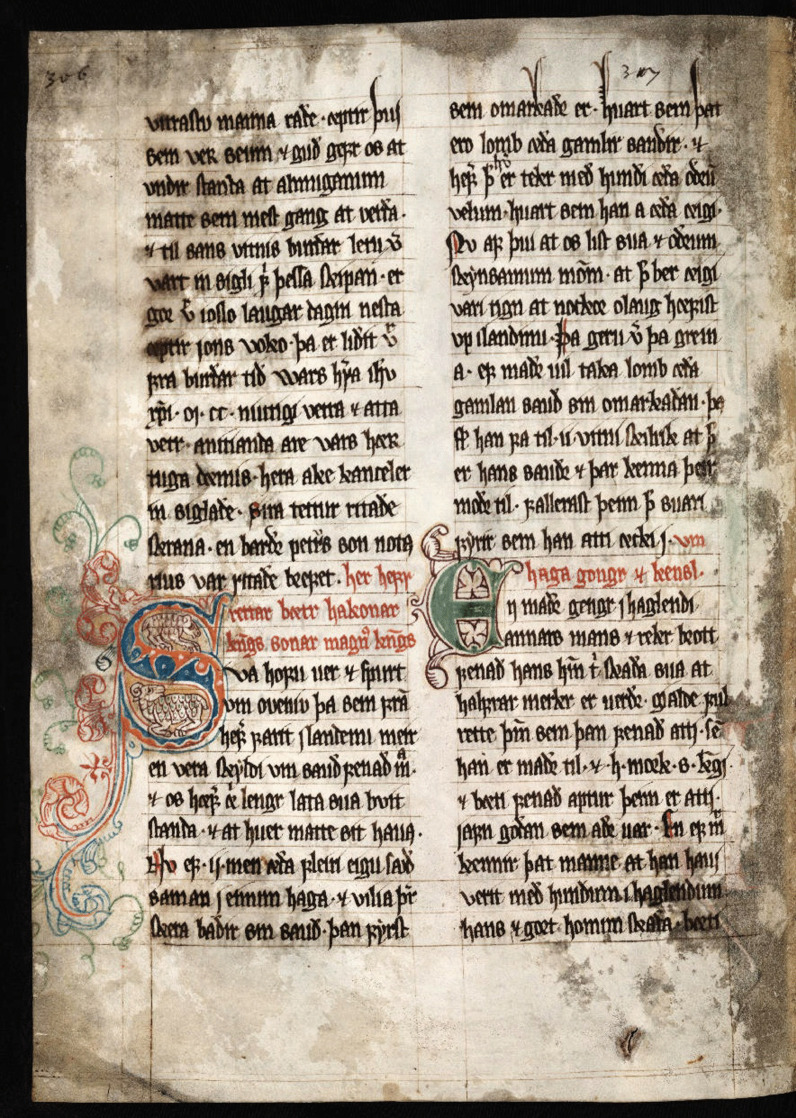
Page from the *sauðabréfit* ‘sheep letter’ (*Seyðabrævið* in contemporary Faroese) from 1298, the oldest surviving written document from the Faroes. It deals principally with sheep husbandry. This page is from the copy of *Lundarbókin* (Book of Lund) with a nice illuminated initial letter with sheep. The manuscript is kept in the University Library of Lund in Sweden. The National Archives of Tórshavn own another copy of the manuscript

A Royal Decree from 1298, known as the Sheep Letter (*Seyðabrævið*) [40], deals with sheep husbandry [41]. According to this decree, the islanders are allowed to keep 70,000 adult sheep during the summer season. Noteworthy is that the figure in question has remained virtually unchanged since 1298 (Fig. 4). In fact, a greater number would lead to overgrazing with the subsequent risk of soil erosion [42]. About 40,000 sheep are slaughtered each year. Thus, we may observe that the Faroese local diet and food system has a tradition of more than 1000 years [43].

Deep knowledge about the environment has been of great importance for sheep husbandry on the islands. The Faroese ethnologist Robert Joensen writes that when he, after a few years away for higher education, returned to his native islands, he was convinced that he now possessed a knowledge that was superior to that of the village people who had only limited or almost no school education. However, it soon became clear to him that many of them possessed a great knowledge of things of which he knew nothing which they had had acquired through daily work in the fields, in the mountains, and on the sea. Joensen wrote that he ‘began to understand that their knowledge was not less than mine, even if it was different’ [44, p. 13]. For Joensen, this discovery became the prelude to an outstanding practice of documenting old folk knowledge. He found that the Faroese had a very detailed knowledge of, for example, the sheep's habits, anatomy, and characteristics that had been a prerequisite for sustainable utilization in older times [44].

Sheep husbandry is conducted in almost the same way today as it has always been. The local sheep is short-tailed, small and a very hardy breed. The animals go out all year round. There are still small horseshoe-shaped shelters, *ból*, with a wall at waist height, intended for about 40 sheep, to be seen in the landscape. However, sheep only go there in winter during snow and storms. Nowadays, the shelters are sometimes equipped with roofs. The sheep flocks graze in the outfield and high up in the mountains. In wintertime, the sheep graze in the lower areas or in the infield. In the 1930s, shepherds still tended the flocks, but nowadays the sheep are left alone in the mountains. Even if he does not follow the flock as the shepherd did before, nowadays a *seyðamaður* (sheep man) regularly supervises the sheep in the outfield. Some sheep are placed on steep cliffs, so-called *feitilendi* (fat pasture), which is said to yield better meat because of the hardships endured while grazing [45, 46]. Sheep are threatened by many dangers. They get lost or freeze to death; in fog or caught by snow blindness they fall into the sea; sometimes they are blown into the sea. Ravens, *Corvus corax*, were earlier believed to cause the death of many lambs born in the mountains [47].

Although sheep farming nowadays plays an insignificant role in the economy, it is still of great cultural and social importance [2, 36]. All mutton production depends on home slaughtering, which usually takes place in the villages. The slaughtered sheep still provide hides, blood, meat, offal, and tallow. Also, the head is boiled in water and simmered for a couple of hours until almost everything, except the bones, can be eaten. It is also eaten *ræst*, i.e. having undergone fermentation [2, 48].

The production of edible tallow is generally considered women’s work and those who possess this wisdom maintain intricate knowledge of the sheep’s anatomy [49]. They know which kind of tallow should be used when preparing various kinds of food. Black pudding, *blóðmørur*, made with sheep blood, flour, and tallow is eaten with treacle. Many more dishes are made using either sheep suet or tallow [1, 2, 50].

Offal is important: liver, heart, and kidney is eaten and made into various dishes. One way of preserving mutton is to sprinkle the boiled meat with salt. This kind of meat, known as *skinsakjøt*, is regarded as festive food. In the local cuisine, some very old-fashioned dishes have survived up to the present day [51].

## Dried and fermented mutton

Nowadays, when most people have access to freezers, mutton is commonly eaten fresh. Imported frozen lamb meat can be bought in the local grocery shops. It is popular as a Sunday roast, known as *lambasteik*, served with boiled potatoes and gravy. However, fresh mutton can, of course, be served in a number of different ways. Faroese cookbooks provide various recipes [50].

The population as a whole still knows how to appreciate fermented meat as both festive, and occasionally as everyday food [52]. The various fermented and dried kinds of mutton are still primarily produced in the home, and usually through a combined effort of the husband and wife.

The most common way to preserve animal products before home freezers became common in the late 1960s and early 1970s was customarily through drying and fermentation. Mammal meat, birds, and fish were wind-dried and no salt was used in the process. The Faroe islanders had to dry all their food to preserve it (since the trees disappeared already in the Middle Ages, it has never been possible to smoke the meat) [4, 5, 53]. In the folk taxonomy, there are three phases in the drying process. Each phase gives the mutton to a different consistency, scent, and flavour. Weather and temperature are crucial for the drying process. In October, when the process is beginning, the average temperature is about 7 °C (although it can range between – 5 °C to + 13 °C). Cold weather in the beginning of the process will affect the taste negatively, and high temperature brings the risk that the product will be inedible and even dangerous to eat.

Both aerobic and anaerobic microorganisms seem to be important in the fermentation process. Various bacteria—*Staphylococcus*, *Flavobactericum*, *Micrococcus* and Enterobactecraiceae—have been identified among the aerobic microbiota on the surface of the meat. Yeast biodiversity identified on the surface of the dried mutton reveals *Candida famata* (F.C. Harrison) E.K. Novák & Zsolt, 1961, *Candida membranifaciens* (Lodder & Kreger-van Rij) Wick. & Burton, 1954, *Trichosporon mucoides* E. Guého & M.T. Sm., 1992 and *Rhodotorula glutinis* (Fresen.) F.C. Harrison, 1928. The identified moulds were *Cladosporium*, *Penicillium* spp. and *Mucor* [54]. The microbiota on the fermented mutton is still rather underexplored, However, further knowledge of the microbial composition of *skerpikjøt* has recently (2023) been published by a Belgian-Danish-Faroese research team [55].

The process from slaughtering to complete dry meat takes three to five months. The first phase is called *visnaður*, which occurs after just a few days. However, lamb is not eaten after such a short period. The second stage is called *ræstur*. It is of less predictable length, and provides dried meat suitable for eating cooked. Usually, it occurs around Christmas. The meat is fermented and semi-dry. *Ræst* meat has a very distinctive, strong flavour, which is appreciated by the islanders as a highly valued delicacy. The final phase provides the most sought after and valued fermented meat, known as *skerpikjøt*.

*Ræst* meat has a typical sharp and pungent taste, which is often mistaken by outsiders for being rotten. In fact, it is fermented with the help of lactobacteria. In 1846, the Danish doctor Peter Ludwig Panum observed the food habits of the Faroe islanders. In addition to a kind of soup made of fat, small pieces of dried meat and barley grain, Panum reported that they ate:

‘… *ræst,* that is, half-spoiled meat […]. The same method of preserving meat which is used for lamb is used also for pilot whale meat, fish, or bird meat; all are hung up to dry without any preparation by salting, smoking, or the like. In the course of several months, when the meat […] is neither fresh nor wind-dried, it is called *ræst*, a word that can be translated by no other term than ‘half rotten,’ an epithet fully merited by this meat, considering the abominable odour it spreads, its unpleasing, mouldy appearance, and it’s not infrequent occupation by maggots’ [56, p. 288].

**Fig. 5:**
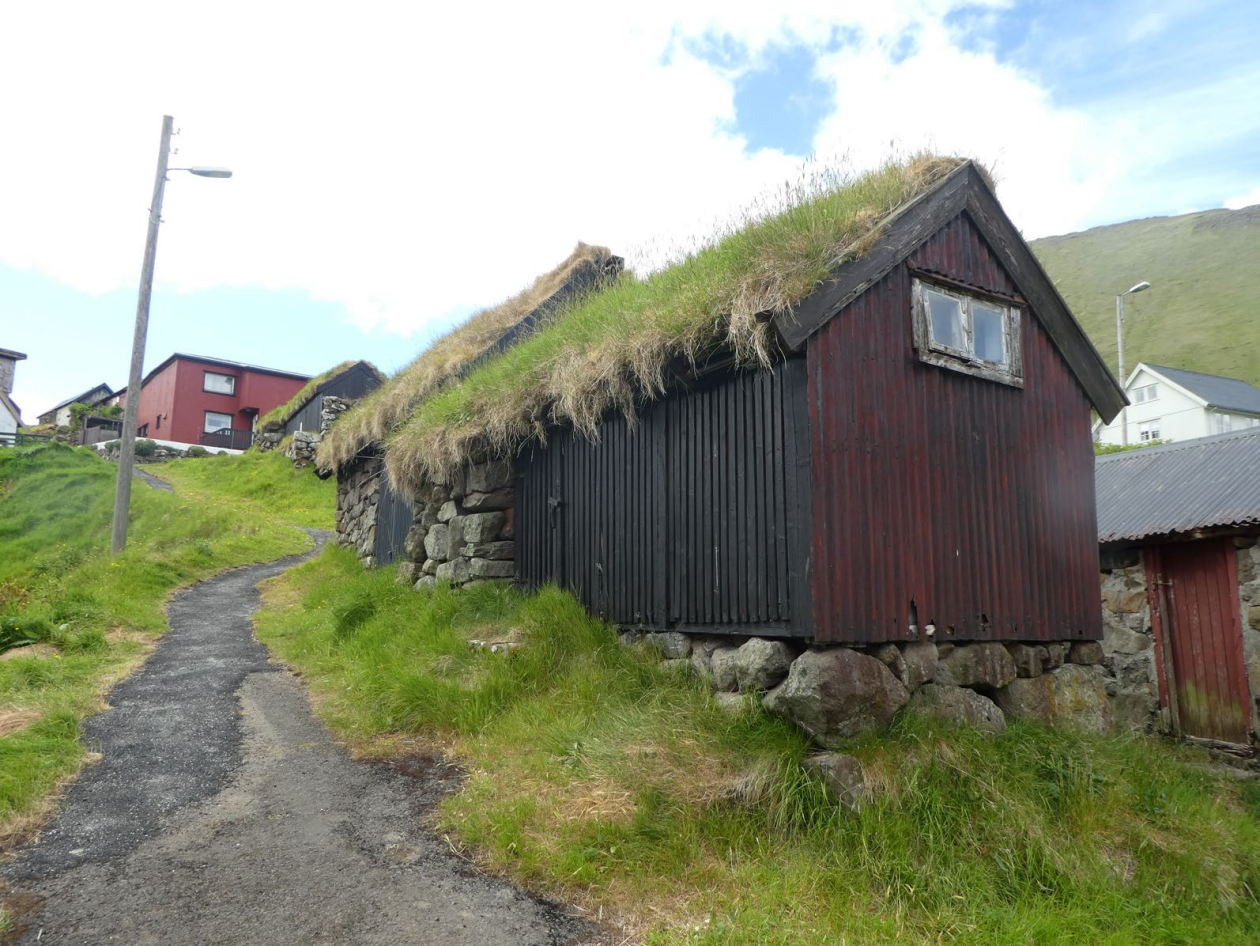
Traditional *hjallur*, a shed ventilated by the wind that is used for wind drying and storing mutton, Mikladalur, Kalsoy (Photo Ingvar Svanberg, 2017)

The politician Edward Mitens remembered, from his childhood in the village Tvøroyri in the late nineteenth century, that most animal food products were hung up and eaten either as *ræst* or dried [57]. *Skerpikjøt* is preserved in specially built wooden outhouses, *hjallur,* which have sides composed of vertical slats that allow space for wind to blow in from the sea [2, 5, 36]. Devoted consumers claim that quality differences in taste of the dried, fermented mutton vary from island to island, and from villages to villages, depending on wind and weather conditions, as well as grazing conditions for the sheep (Fig. 5).

Fermented mutton is usually considered safe to eat. However, there are some health risks connected with drying the mutton, especially nowadays when not everyone has access to traditional *hjallur*, or has mastered the old established knowhow to correctly prepare the meat into *skerpikjøt*. These circumstances are risk factors for the meat to develop foodborne botulism. This potentially fatal disease is caused by a neurotoxin produced by the bacteria *Clostridum botulinum* van Ermengem, 1896. Between 1988 and 2007, nine cases of botulism were reported from the Faroes, two with a fatal outcome. It is believed that fish products contaminated the mutton [58].

## Discussion

Locally available resources through the mid-twentieth century logically dominated Faroese food habits. A health and odontology survey in 1937–38, during the Great Depression, found the nutritional status of the otherwise poor villagers living on a fish and fermented meat diet adequate from a health perspective [59]. The birth weight was higher than elsewhere in the Nordic countries [60]. The seasonal availability of fresh and/or dried-fermented food persisted in the Faroese villages until the 1960s [1, 4, 5, 61].

The ecological conditions and the relative isolation in the mid-Atlantic have meant that animal products have, and still do, dominate time-honoured traditional food culture. Cereals and vegetables played a minor role in the traditional cuisine; potatoes and other root vegetables were the most common. Food availability was seasonal with periods of fresh food alternating with periods of mostly dried and fermented food (other ways of food preservation did not existed). The island communities were characterized by a storage economy up to the early twentieth century [2, 5, 36, 61].

**Fig. 6:**
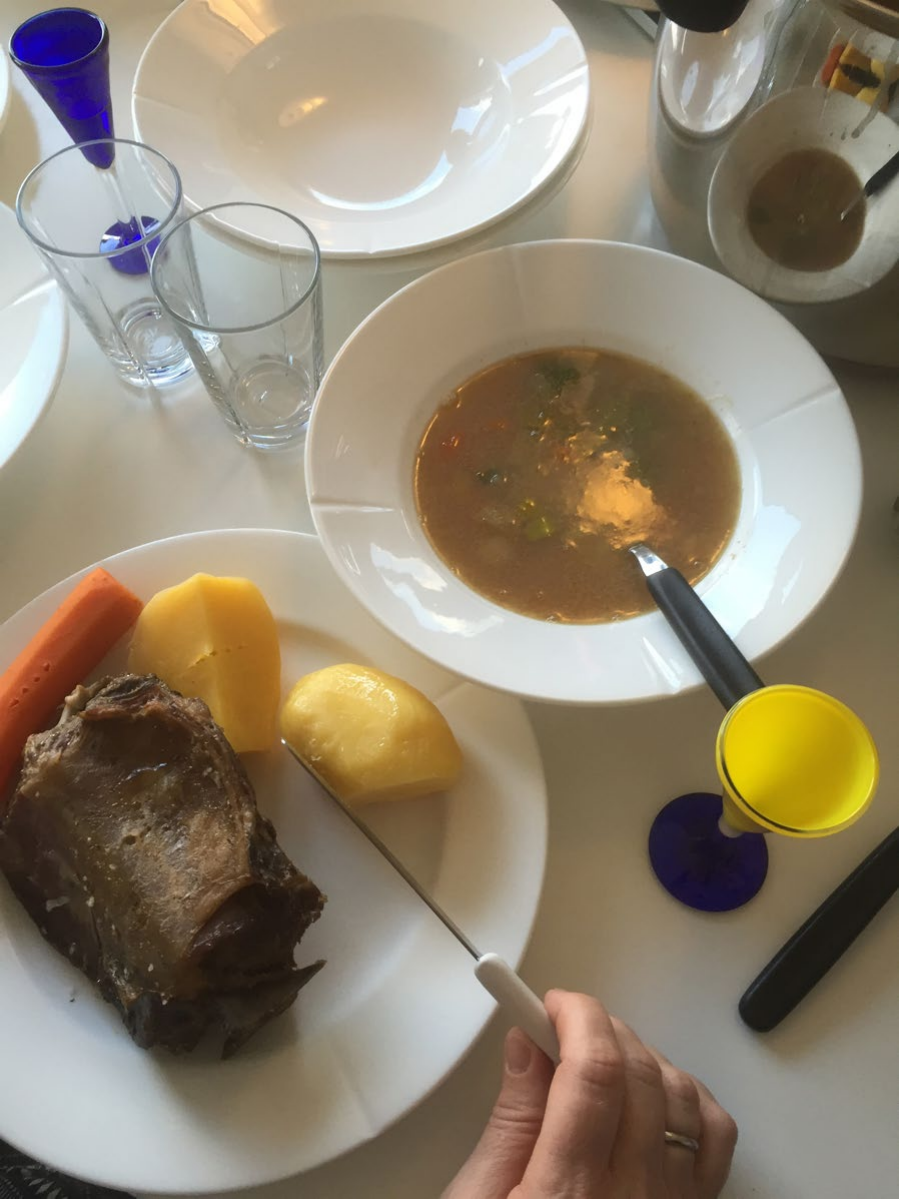
*Ræstkjøt* eaten with root vegetables (Swedish turnip, carrots). The broth is served as a side dish to dip the mutton, potatoes and roots (Photo Osva Olsen, November 2016)

**Fig. 7:**
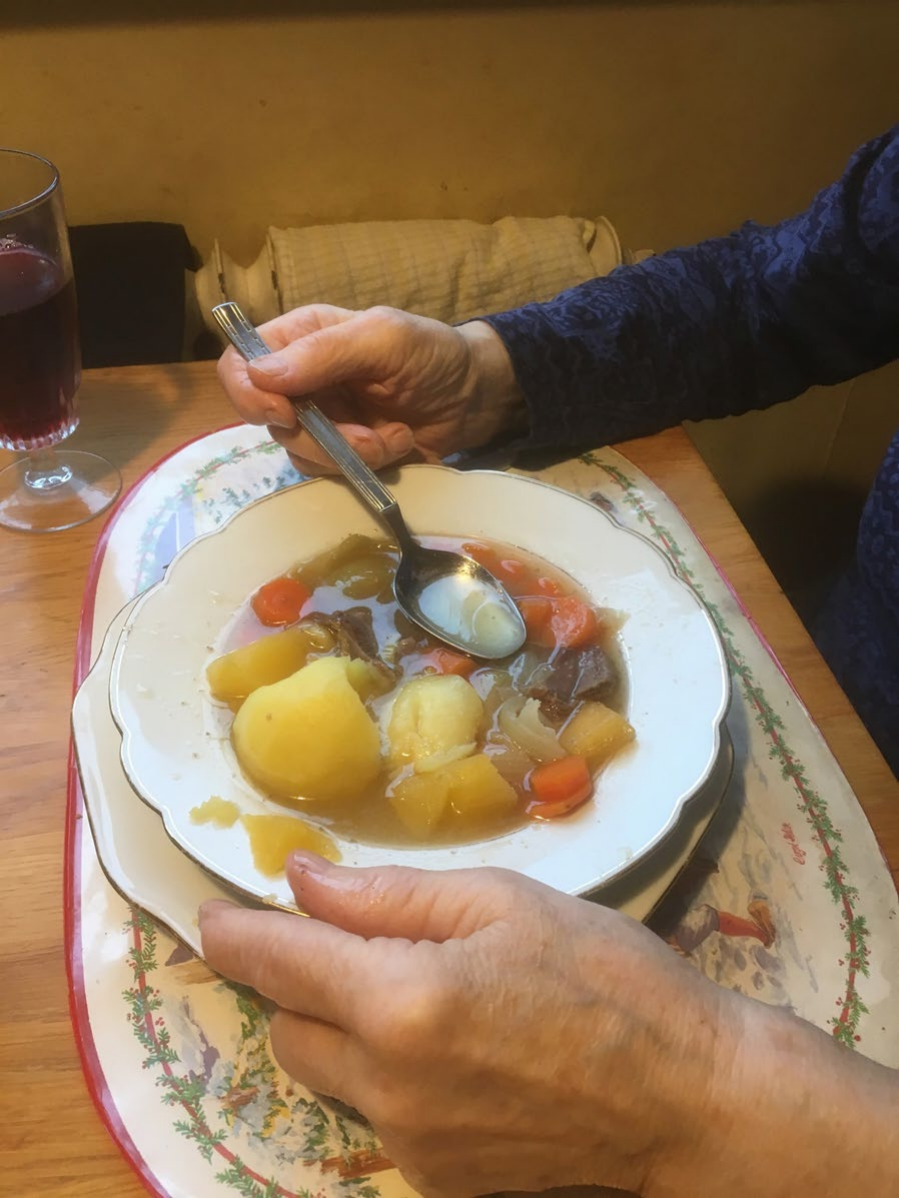
Soup made of broth, potatoes, leeks and *ræst* mutton (Photo Ingvar Svanberg, December 2019

The elderly generation continues to consume a diet based on fish and meat. Many locals are proud that several aspects of their food tradition have been preserved. The islanders themselves produce significant quantities of traditional food items [1, 2, 5]. Although the share of private food production (fishing, hunting, and animal husbandry) is still important, an increasing amount of food is obtained from the international food market. According to recent estimates, the self-sufficiency degree in the Faroe Islands is 22 per cent [62–64]. These subsistence practices guarantee the survival of traditional food knowledge in the Faroes (Figs. 6–7).

**Fig. 8:**
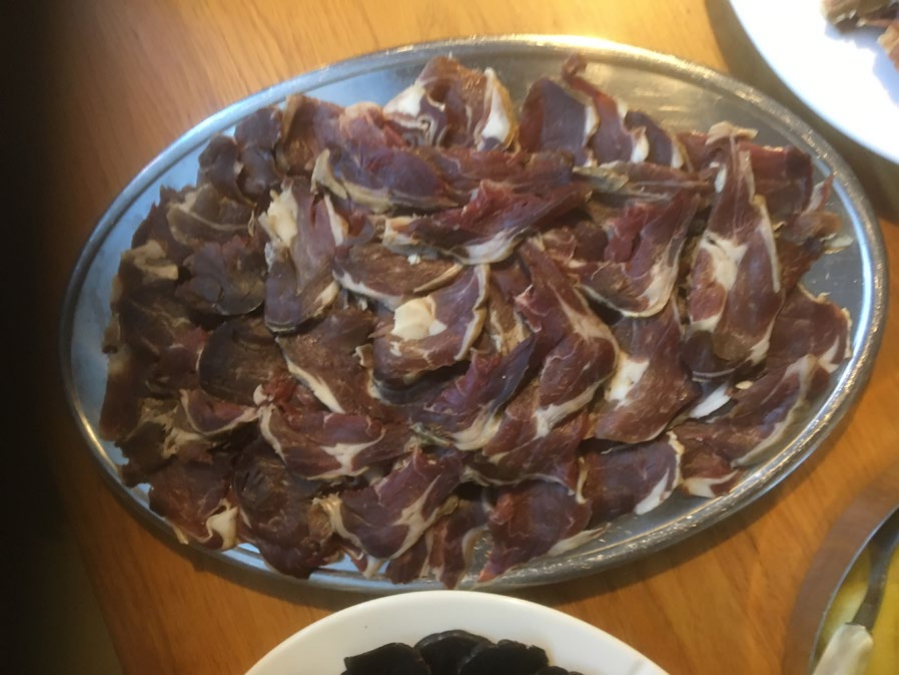
Slices of *skerpikjøt* served at a festive buffet table. It is usually served with rye bread, made of sourdough, for *náturði* ‘evening meal’ (Photo Ingvar Svanberg, December 2017)

Today, the urban middle-class has a growing interest in gourmet food [1, 61]. There is now a demand for dishes made exclusively from local ingredients, thus following the general European trend. The ‘New Nordic Cuisine’ has attracted a great deal of interest internationally in recent years [64, 65]. This also applies to the Faroe Islands, where gastro-tourism for food trotters has become increasingly popular. The islands have seen a strong influx of tourists in recent years. The tourism industry experienced only a brief downturn during the Covid 19-pandemic. Currently it is growing like never before, and the Faroe Islands is seen as a culinary destination by many [1, 66, 67]. One reason for this is the exclusive avant-garde restaurant KOKS that has made local food its trademark. For that, it has been rewarded with 2 Michelin Stars since 2017. Fermented meat may be difficult to render in various forms, but KOKS (in 2023, temporarily relocated to Ilimanaq Lodge, Greenland, pending the completion of new premises in the Faroe Islands) has succeeded in doing that and transformed it into modern food. In a time when industrially produced food is eliminating sensations of taste and smell, fermented products with a distinctive accent are attracting food lovers from all over the world [1, 68]. An interesting newcomer to the gastro-tourism scene in the Faroes is an institution known as *heimablídni* (home hospitality) where visitors can enjoy dining experiences in people’s homes. Here the tourists are treated with locally produced home-made food (for instance roasted lamb, cold cuts (*kjøtpylsa*, *rullupylsa*) made of mutton and tallow, salmon, fillet of cod, home-baked rye bread, rhubarb dessert, locally produced beverages), sometimes including *skerpikjøt,* while being entertained by their hosts with storytelling [1, 69] (Fig. 8).

Gourmet restaurant food is not for everyone, and it is not an everyday food. However, there is an interesting newcomer among street foods in the Faroes. Since 2018, *ræstkjøtaburgari*, that is, a kind of meat sandwich made of *ræstkjøt* served with shredded red cabbage, is available from a mobile food stall, which moves around the islands and makes the fermented mutton available for both tourists and hungry islanders alike. Otherwise, there is an increasing preference among the young to eat less healthily, and from a taste point of view, far blander industrial food [1]. This is especially true for the urban population, which has to rely on the relatively large import of food from abroad. This is despite the fact that the Faroe Islands is one big pantry, and largely lives by exporting food (fish, shellfish) to the rest of the world [2, 63].

**Fig. 9:**
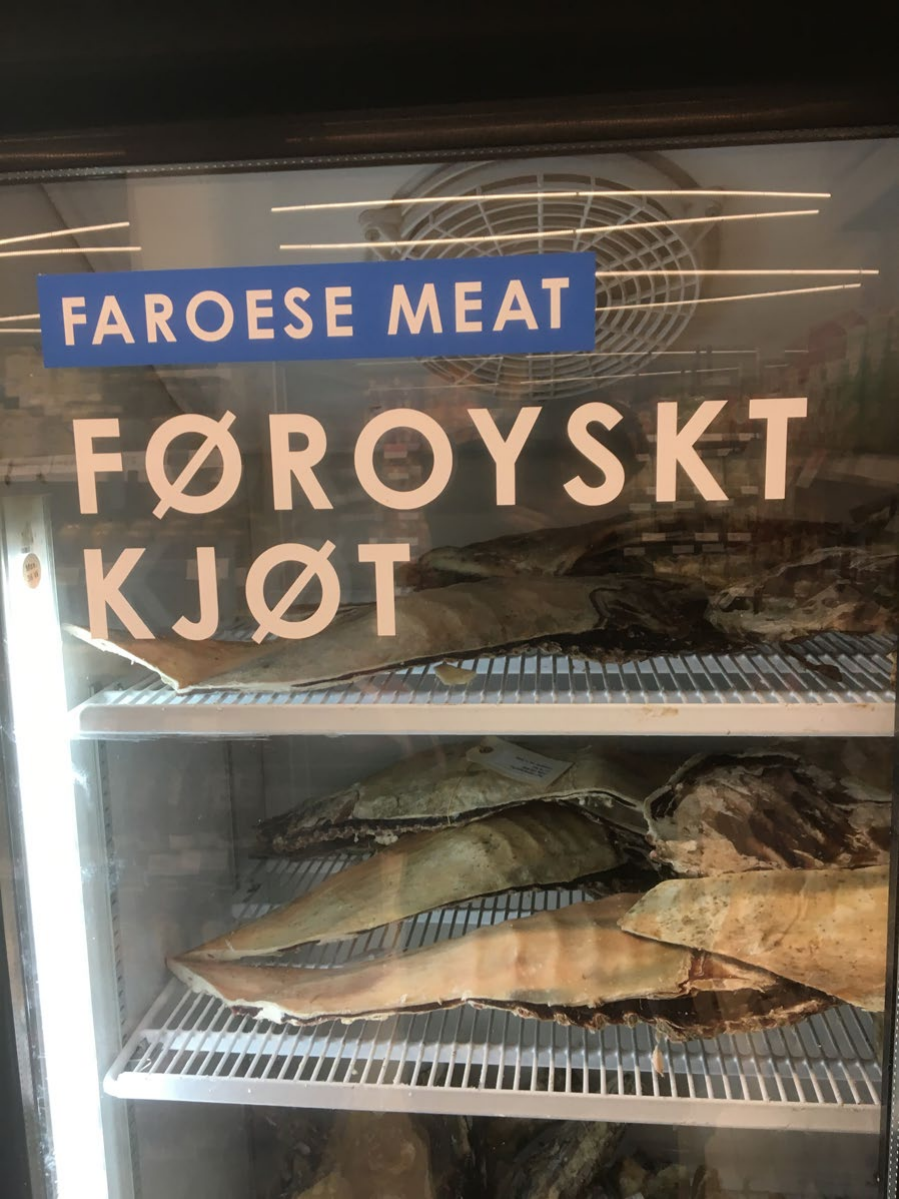
Locally ripened sheep legs (*skerpikjøt*)—an exclusive food item available in a grocery store in Tórshavn just before Christmas (Photo Ingvar Svanberg, December 2022)

To increase interest in locally produced food, small grassroots initiatives are present. The global movement Slow Food is active in the Faroes (Slow Food Føroyar). Another interesting initiative is called Matkovin (The Pantry), a website launched by a food activist and ethnologist to shorten the distance between the Faroese consumer and the Faroese producer. The goal with this online infrastructure is to create ties between producers and consumers in order to make local products available. Recipes can be shared, contact and trust between producer and consumer is created and food can be ordered through this digital platform. There are a few farmers producing *skerpikjøt* and *ræstkjøt* that are involved in this network [63]. This organization, will enable producers to reach a domestic, urban clientele of consumers who would otherwise not have a chance to obtain such meat. Eating Faroese food is a question of access to healthy and organically produced food. By the process of fermentation, it has acquired its own particular taste, which is highly appreciated by both food connoisseurs and most locals. Through this new network, urban people and professionals, with no close relatives in the countryside, can still enjoy fermented mutton, at least if they can afford it (Fig. 9).

Over the generations, the relative isolation of the Faroe Islands has reinforced a strong cultural identity and a reliance on local foods. The strong connection between cultural identity and fishing, and indeed the island’s food security, continues to be expressed today [1, 2, 53, 69, 70]. Meat, especially mutton, plays an important role in the Faroese cuisine and is still of great importance in the islands’ foodscape. Sheep husbandry is also important to maintain biodiversity in the Faroese landscape. Furthermore, sheep are significant symbols of identity that are found in many contexts in Faroese society [16, 36, 71].

Fermented mutton remains a unique foodway and important part of the diet for many islanders. It is indeed a heritage food that has been perpetuated for centuries on the Faroes, probably since the Vikings settled there. Of course, there are individuals whose diets are characterized by modern, imported, processed factory foods and who have not learned to appreciate the special taste that fermented meat has, nor do they like the intrusive smell when and after eating the meat. During the roughly 30 years that I have been able to follow Faroese dietary habits, there have been rapid changes. In fact, most food available in the rather few grocery stores and supermarkets is imported [2, 61]. In 1990, the Faroe Islands imported for 227,555,200 DKK of foods, beverages, and tobacco (classified according to UN Trade Statistics as Broad Economic Categories); in 2000 for 346,158,200 DKK; and in 2022 the figure was 895,611,700 DKK. 1 DKK is 0.15 USD [19]. However, these figures say very little about the actual local food consumption.

Processed food bought in grocery stores has become increasingly common as everyday food, especially among a younger generation [2]. Health-conscious young urban Faroese islanders naturally consume modern factory-produced and imported fermented food products (e.g. dairy products, kimchi, kombucha, sauerkraut, etc.) available in Tórshavn’s supermarket, items popular for the health benefits that they can provide [72].

The meat consumption is still high on the Faroes, mutton is preferred and most lamb meat consumed is nowadays imported from abroad, and there are of course many ways to prepare it [73]. However, it is usually eaten as a rather simple dish: *lambasteik* i.e. ‘lamb steak’ served with potatoes and boiled imported vegetables (carrots, green peas), although various recipes are available in the cookery books used by the Faroese households [50, 74]. However, traditional wind-dried and fermented fish is still eaten as a daily food despite its intrusive small by many families, especially by rural households. Sourdough bread is staple food, often eaten with *skerpikjøt*. Other traditional home-made fermented animal products are also readily available. There is no sign that the contemporary economic changes will challenge the interest for Faroese heritage food such as fermented fish and mutton. Appreciating and consuming locally produced food is not only a question of taste preferences but also an important way of expressing Faroese cultural identity [1, 2, 5].

Locally produced fermented fish, mutton and other animal products contribute to a sustainable food system and a diversified food production. It also procures safety and security for the rural communities on the Faroe Islands [1, 5]. The way of subsistence affects the landscape and biodiversity including the microbiota, and thus the entire Faroese foodscape [75]. However, a future threat for the survival of the fermented mutton in the local cuisine will be global climate change, which will have consequences for the very basis of Faroese society. Traditional sheep husbandry is at risk as pastures and their vegetation change due to warming. However, there is no research on this yet. Warmer autumns will also make it more difficult or impossible for the natural fermentation process to take place. This is probably a greater danger than any other factor to the survival of traditional foods on the islands [76].

## Conclusions

This article has investigated the production of air-dried fermented mutton in the Faroe Islands, which is a unique food culture. Among the Nordic countries, the Faroe Islands are the only location where fermented mammal meat continues to be used as food. It is still primarily produced in the homes. The entire process is elaborate, involving sheep husbandry, slaughtering, and fermenting the lamb corpse. Consumption occurs largely within the household, although *skerpikjøt* can be bought in some grocery stores in the capital. Furthermore, this article contributes to the ongoing ethnobiological discussion on the importance of fermented meat products in the traditional diets of northern hemisphere. Although it is a true artisan food which is rather expensive and time-consuming to produce, and it also requires traditional craftmanship that are mastered by fewer and fewer, it has a potential to be an exclusive gourmet dish within the increasing gastro-tourism which the Faroe Islands are currently experiencing.

## Data Availability

The analysed data are incorporated in the research article.
